# Supplementary material for: Binding of single-mutant epidermal growth factor (EGF) ligands alters the stability of the EGF receptor dimer and promotes growth signaling
Source: J Biol Chem. 2021 Jun 11;297(1):100872. doi: 10.1016/j.jbc.2021.100872 (PMC8259408; doi:10.1016/j.jbc.2021.100872)
Supplement: Figures S1–S13; Tables S1–S3; Data S1 [file mmc2.pdf]

SUPPORTING INFORMATION

Binding of single-mutant epidermal growth factor (EGF) ligands alter the stability of the EGF receptor dimer and promote growth signaling

Stefano Pascarelli[1], Dalmira Merzhakupova[1], Gen-Ichiro Uechi[1], Paola Laurino[1*]

[1] Protein Engineering and Evolution Unit, Okinawa Institute of Science and Technology Graduate University, Onna, Okinawa 904-0495 Japan.

* Corresponding author
E-mail: paola.laurino@oist.jp

Table of Contents

Figure S1. DIRpred schema. 3

Figure S2. EGFR ligands orthologs tree. 4

Figure S3. DIRpred statistics. 5

Figure S4. DIRpred score. 6

Figure S5. Crystalized structure of WT EGF with the highlighted position N32, In the interaction with the position Q16 and G18 of the EGFR receptor backbone. 7

Figure S6. The validation of ordered structure of WT EGF and variants. 7

Figure S7. Effects of WT and single-mutant EGF on the receptor phosphorylation and dimerization in A431 cells at additional time points. 8

Figure S8. Effect of 1nM, 10nM and 100nM concentrations of EGF variants on the proliferation of the human normal fibroblast Bj5-tα cell line and mouse normal fibroblast Albino Swiss 3T3 cell line. 8

Figure S9. Effect of 1nM and 10nM concentration of EGF variants on the proliferation of A431 skin cancer cells. 9

Figure S10. A431 cell apoptotic assay. 9

Figure S11. A431 cells apoptosis experiment comparison for No Ligand, N32R, and W50Y samples. 10

Figure S12. Highlight on the EGF-specific residue swapping problem. 10

Figure S13. Details of the mutants and their binding. 11

Table S1. DIRpred score of each position of EGF. 11

Table S2. EGF mutants relative ranking for the DIRpred score. 13

Table S3. ANOVA details. 13

Supplementary data 1. Time-lapse video of the proliferation of A431 and Bj-5tα cells. 15

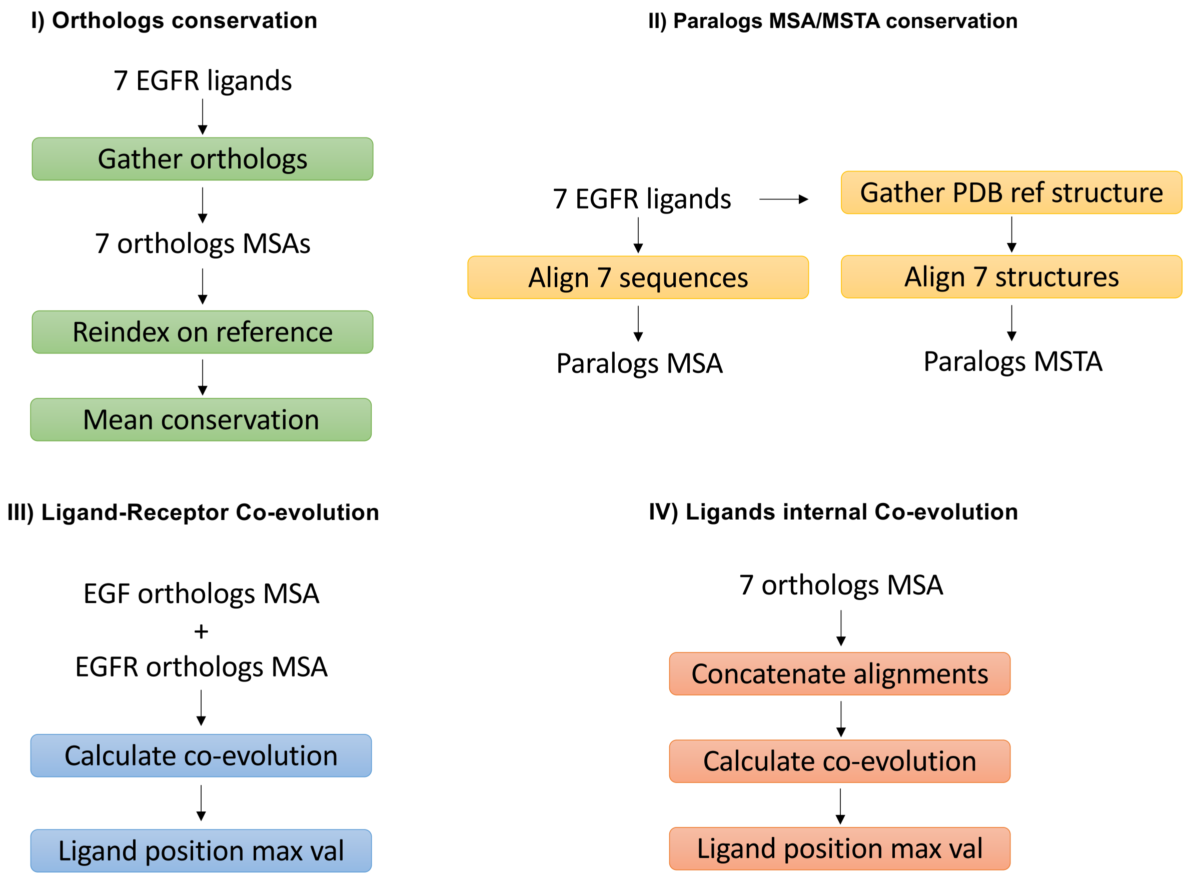

Figure S1. DIRpred schema. A schematic representation of DIRPred, as shown in Figure 7.

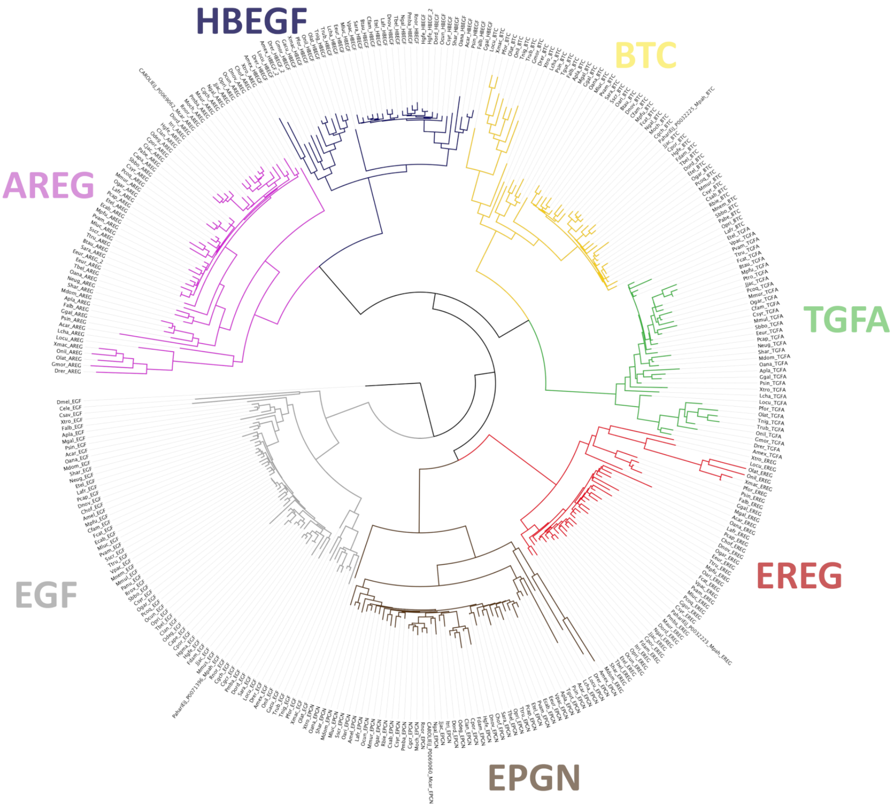

Figure S2. EGFR ligands orthologs tree. The tree was obtained using Maximum Likelihood algorithm. *C elegans* EGF was used as outgroup. The EGFR ligands cluster neatly. Interestingly, fish ligands are found to have longer branches on average. The tree was made using iqtree ModelFinder. The best-fit model used is JTT+R6.

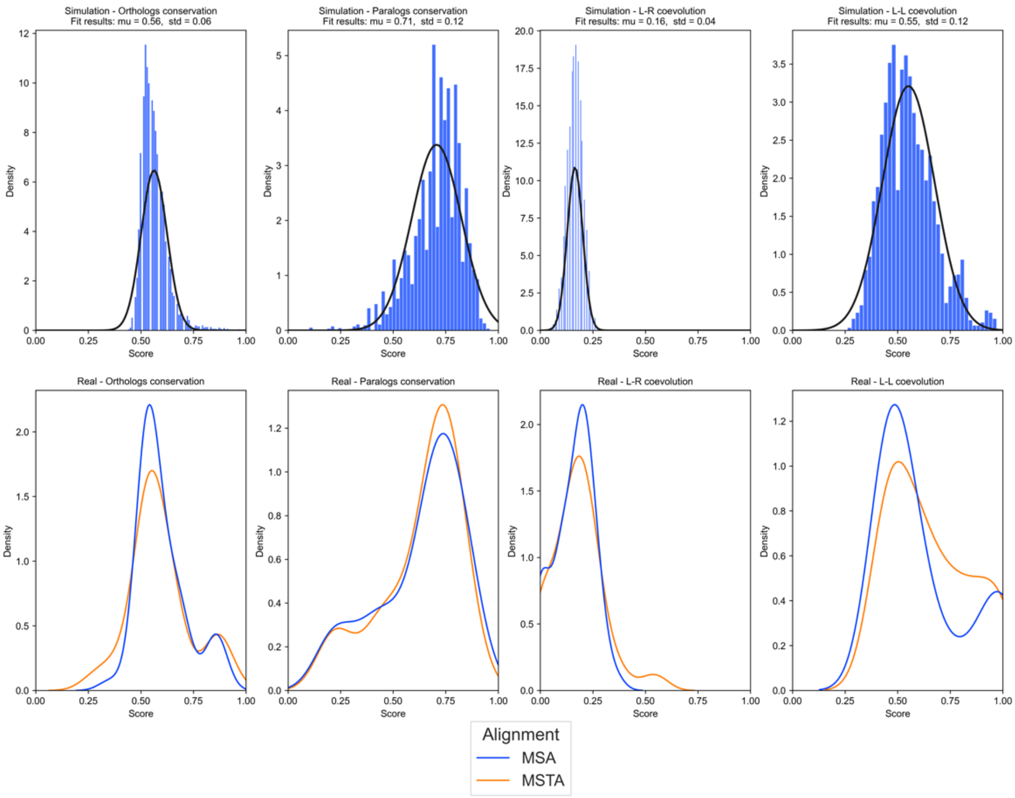

Figure S3. DIRpred statistics. The four columns represent the four DIRpred scores. The top row shows the score distributions of the DIRpred analysis of a dataset of 100 repetitions of simulated evolution. The simulations started from a random 53 amino acid long sequence and were carried out using the WAG model. On the bottom row, the distribution of scores from the real alignments of EGF and its homologs, divided by the sequence or structural alignment seed used for the analysis.

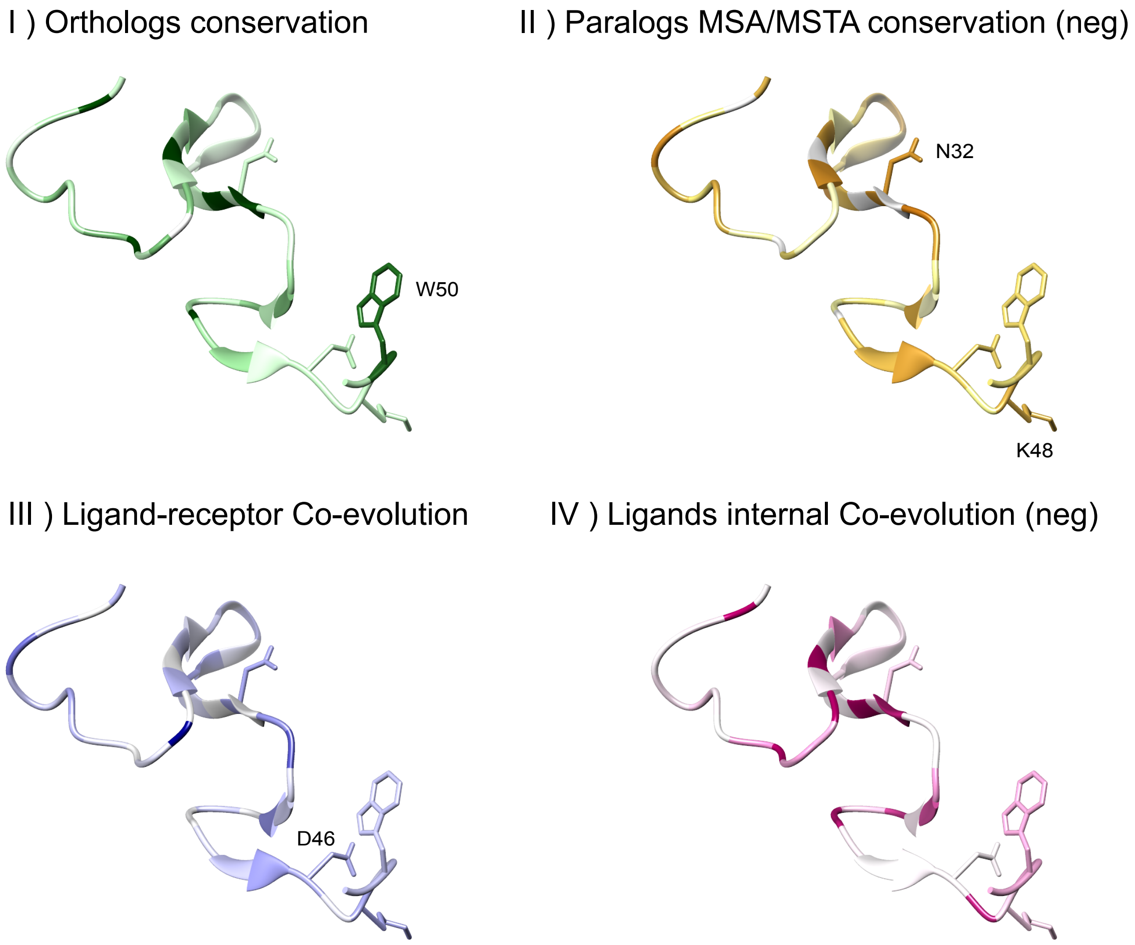

Figure S4. DIRpred score. Structure of EGF (from PDB: 1IVO) colored by the four individual scores. The four sites chosen for mutation are shown in stick representation.

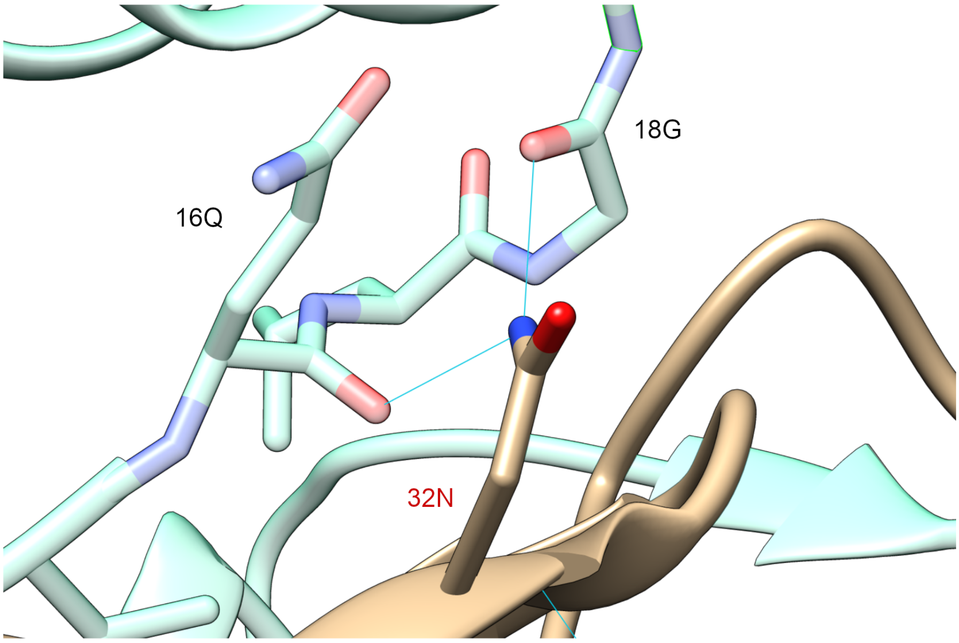

Figure S5. Crystalized structure of WT EGF with the highlighted position N32, In the interaction with the position Q16 and G18 of the EGFR receptor backbone.

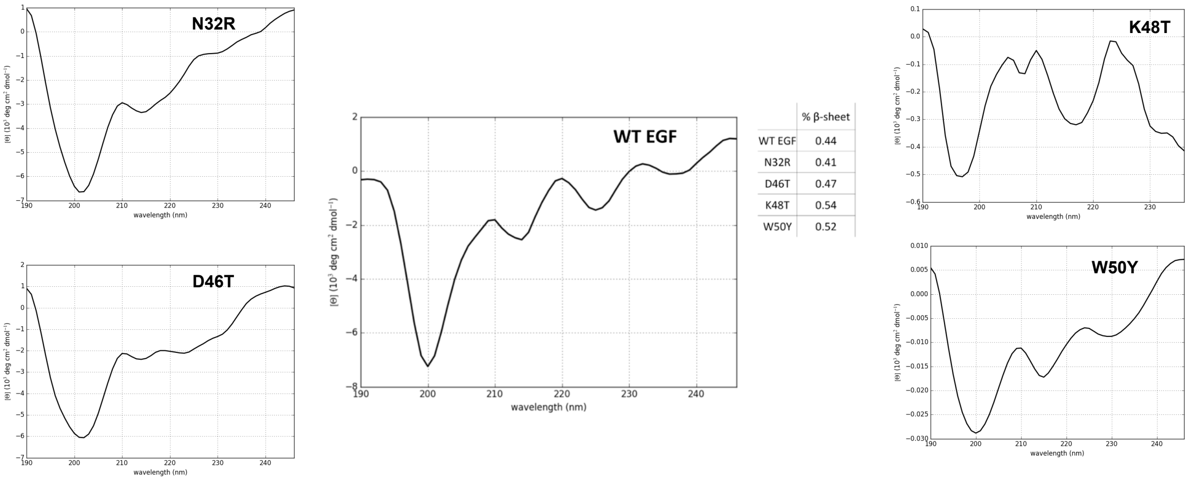

Figure S6. The validation of ordered structure of WT EGF and variants. 0.2 mg/mL of the sample was added to a 10 µm cuvette and analyzed by circular dichroism spectroscopy. All samples showed the presence of a comparable amount of β-sheet. The CD spectrum of EGF was similar to previously published results (26). The plot and analysis was made using CAPITO web tool (27).

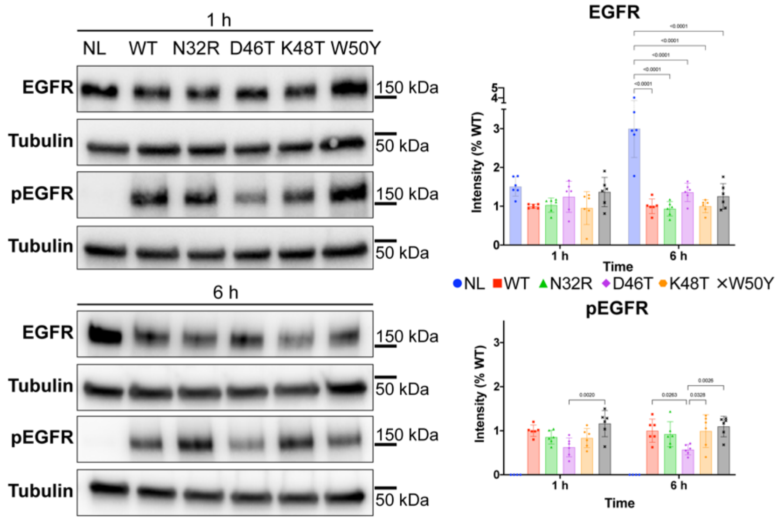

Figure S7. Effects of WT and single-mutant EGF on the receptor phosphorylation and dimerization in A431 cells at additional time points. The effects on the amount of EGFR and the phosphorylation level of Tyr-1173 after treating A431 cells with 100nM concentration of different ligands at 1 and 6 h. The membrane containing EGFR and tubulin was separated after the transfer. The WB was performed in the same conditions as in Figure 4. Data is shown of at least four biological duplicates. The bars represent mean ± s.d. of at least four biological repeats. The number on top of the bars shows the p-values of a 2-way ANOVA multiple comparison corrected for multiple sampling using the Bonferroni correction. Band intensity estimates were calculated using BIO-RAD ImageLab software (BioRad). Plots and statistics were performed using PRISM software (GraphPad). Details of the ANOVA are found in Table S3.

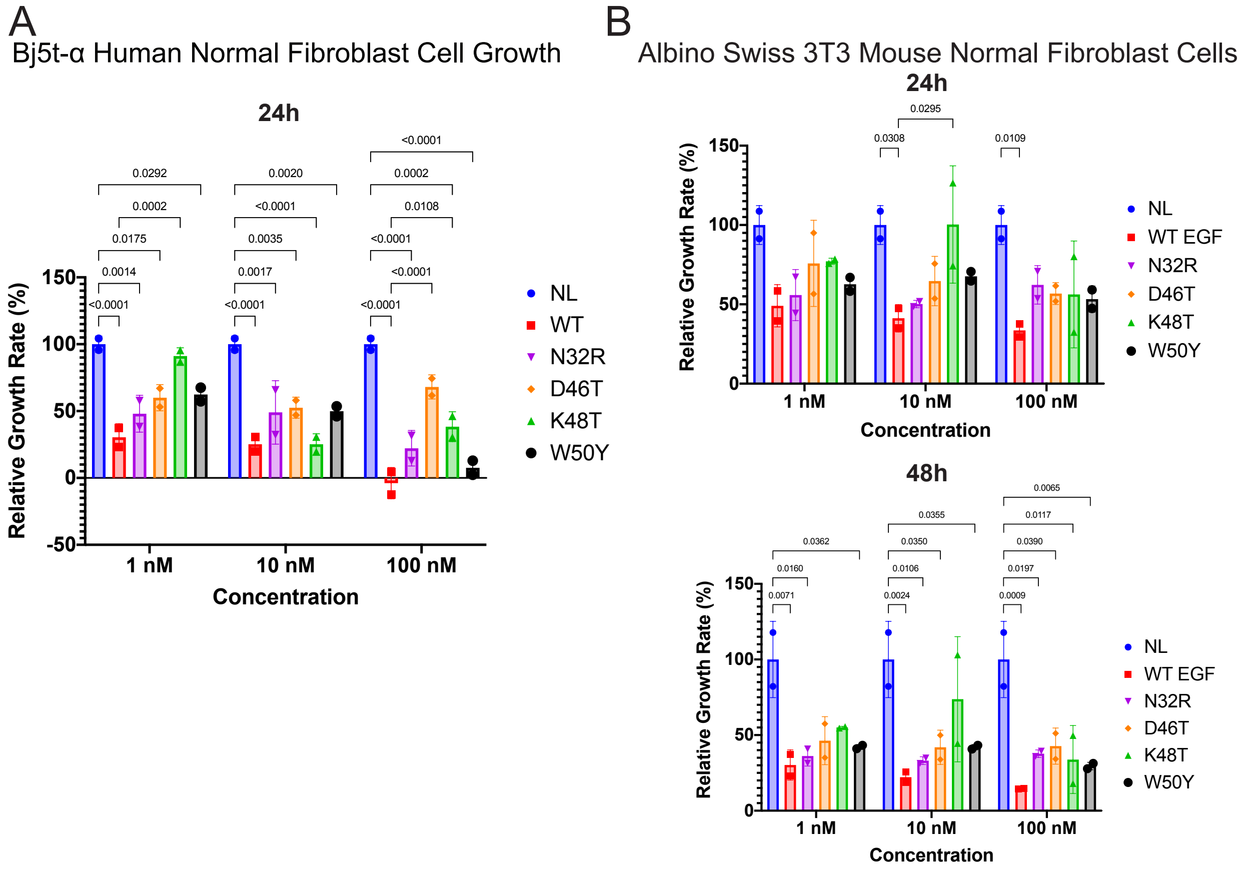

Figure S8. Effect of 1nM, 10nM and 100nM concentrations of EGF variants on the proliferation of the human normal fibroblast Bj5-tα cell line and mouse normal fibroblast Albino Swiss 3T3

cell line. The data represent growth as a percentage of the control based on the confluence of cells (mean ± standard deviation) for each concentration of EGF. The percentage confluence was estimated 24 h and 48 h after the treatment (two replicates per treatment). Details of the ANOVA are found in Table S3.

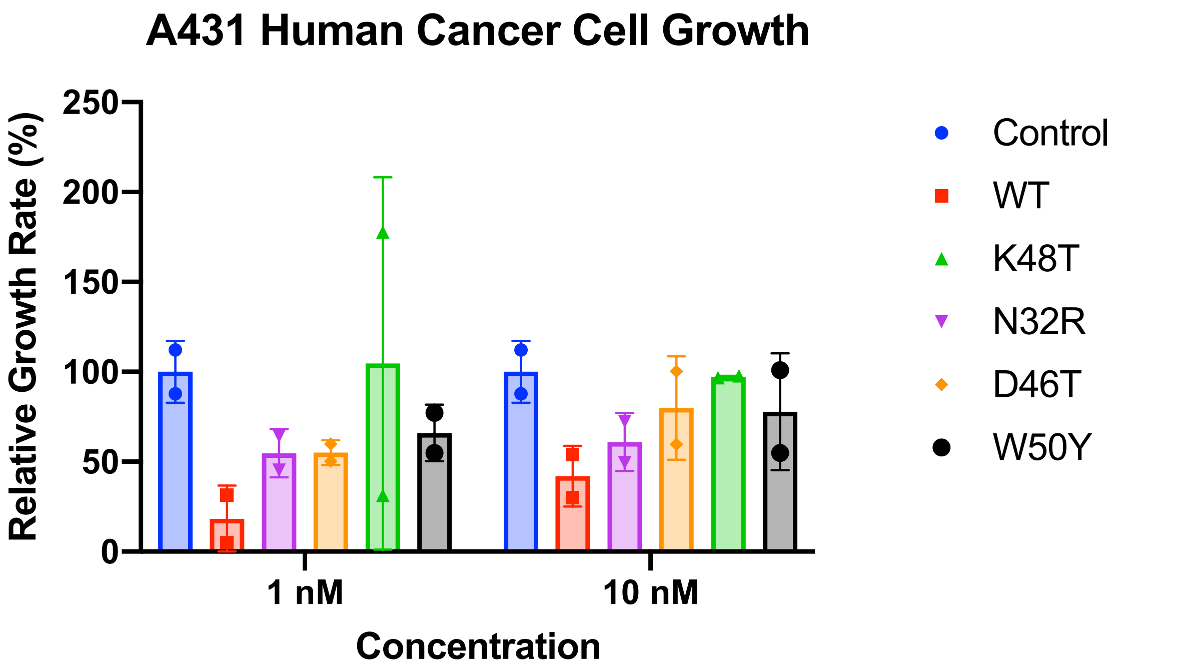

Figure S9. Effect of 1nM and 10nM concentration of EGF variants on the proliferation of A431 skin cancer cells. The data represent the relative growth percentage to control based on the confluence of cells (mean ± standard deviation) for each concentration of EGF variants. The percentage confluence was estimated 48 h after the treatment (two replicates/treatment).

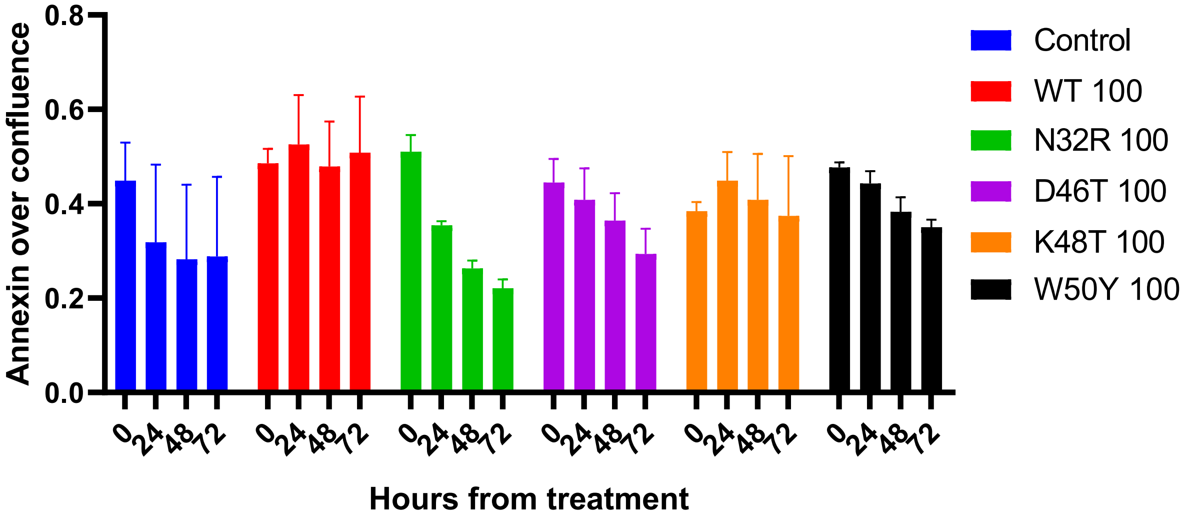

Figure S10. A431 cell apoptotic assay. After 24h growth incubation, A431 cells were treated with saturating concentrations of WT or mutant EGF. The green reagent Annexin V confluence was related to the phase confluence (cell growth). WT EGF is the only sample to show a stable trend after several

days, while all other samples show a reduction in the ratio of Annexin signal to confluence. The apparent high level of K48T could be explained by the change in morphology of the cells.

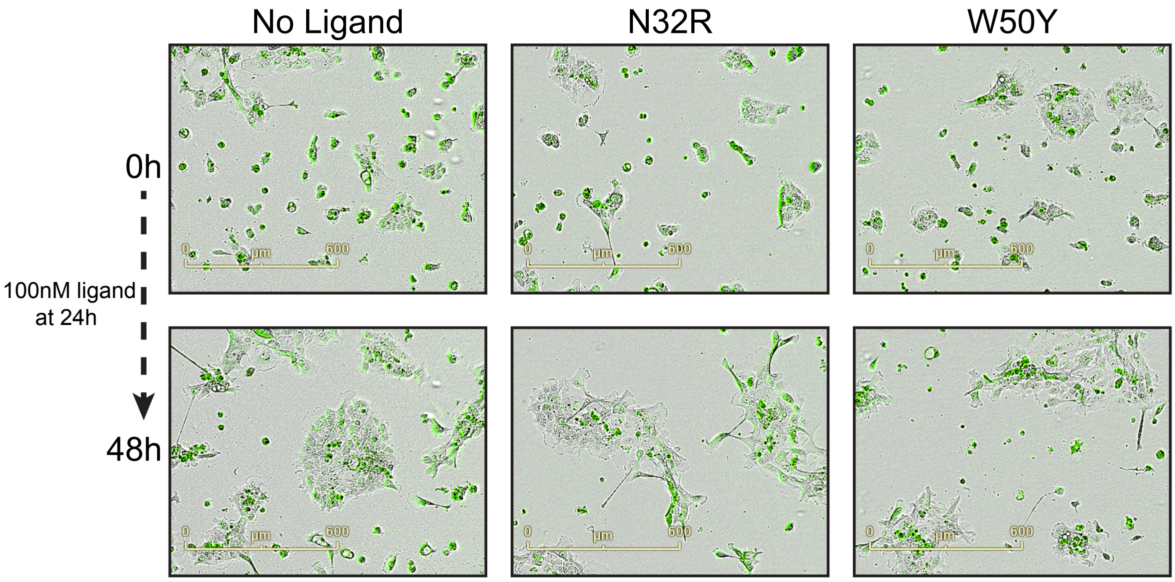

Figure S11. A431 cells apoptosis experiment comparison for No Ligand, N32R, and W50Y samples. Comparison of A431 cell growth after treatment with 100 nM EGF variants N32R and W50Y or untreated. Cells were labeled with fluorescent Annexin V Green Reagent. Plates were pre-warmed prior to data acquisition to avoid condensation and expansion of the plate, which affect autofocus. Images were captured every 2 h (4x magnification) for 3 days in the IncuCyte system.

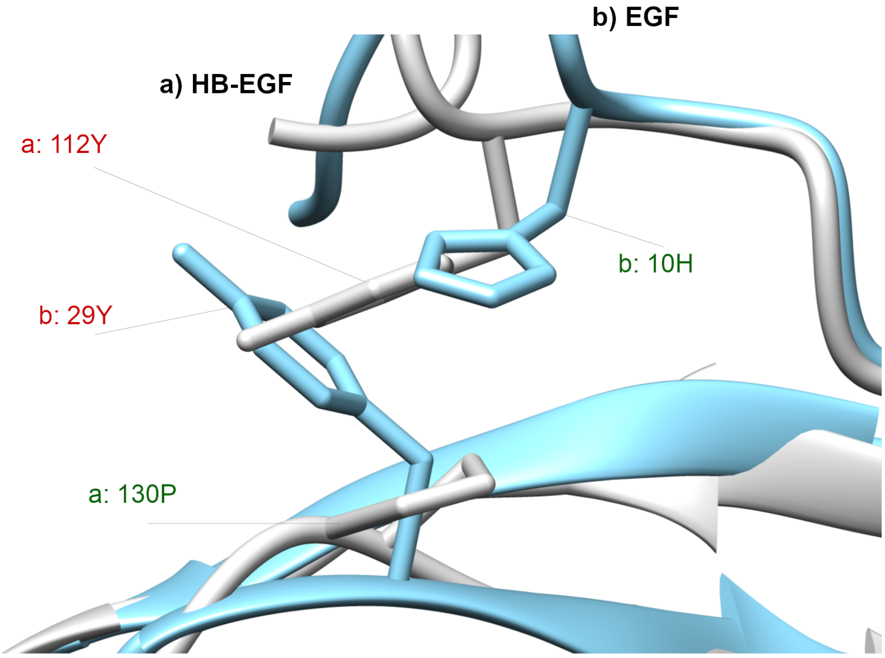

Figure S12. Highlight on the EGF-specific residue swapping problem. EGF is colored in teal, while HB-EGF is colored in white. The tyrosine in EGF position 29 (29Y) is not found on the corresponding

position of HB-EGF. Instead, a proline is found (130P). Though, the interaction between the two residue is probably maintained because, in place of the histidine 10 (10H) of EGF, HB-EGF shows a tyrosine (112Y). When this type of residue swapping is ligand-specific, the lowering of the conservation measures over-estimates the real change happening in that position.

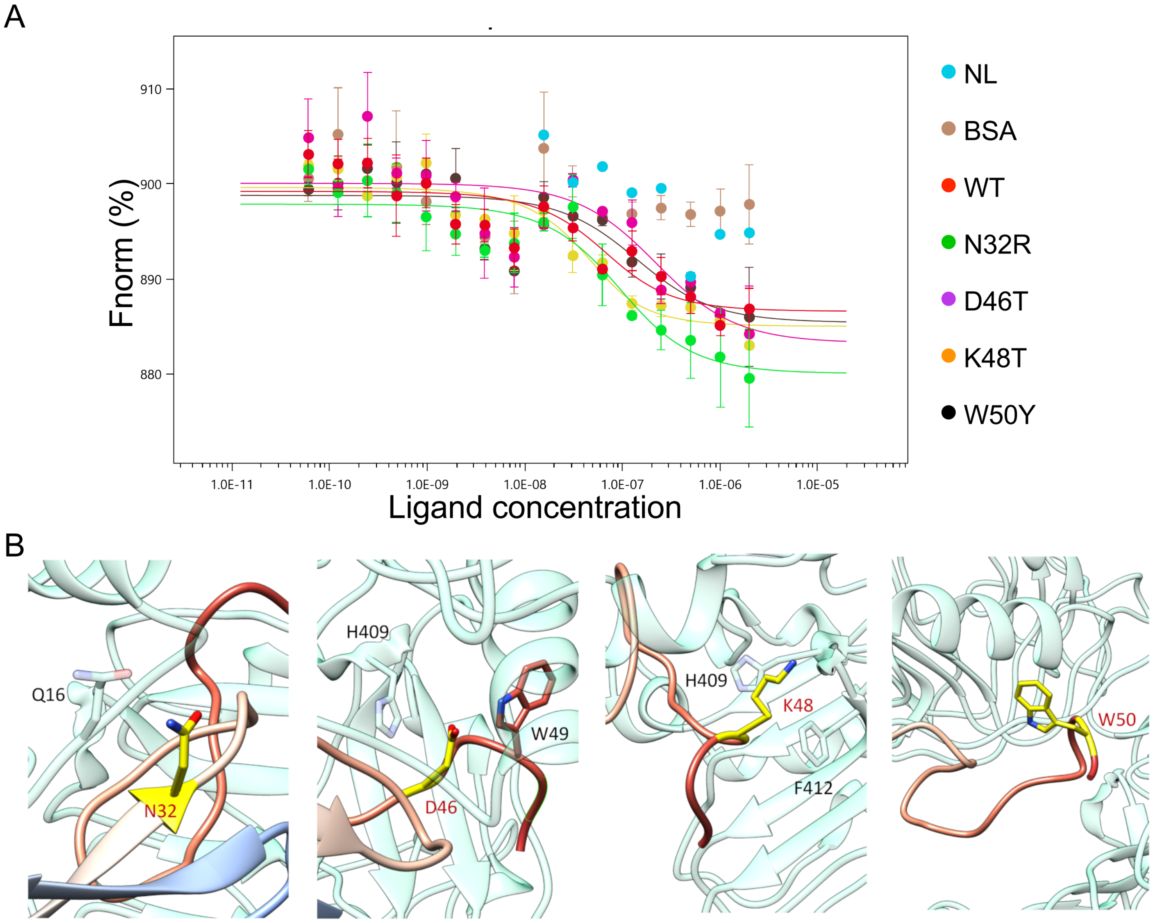

Figure S13. Details of the mutants and their binding. (A) The MTS experiment shown in Figure 3B with an additional No Ligand (NL) sample. The "concentration" coordinate for this sample does not reflect any change in preparation and should be interpreted as a repeat. From this representation, it appears that the BSA response lies within the NL range, while all other ligands show a transition towards higher fluorescent signal at a similar concentration. (B) Zoom-in of the four mutations included in a model starting from PDBID:1IVO.

Table S1. DIRpred score of each position of EGF. The table resembles the actual output of the DIRpred pipeline, reporting the four individual scores and the combined score. Furthermore, we added the P-value associated to each individual score and the sum of P-value ranking.

	POS	ORTHOLOGS CONSERVATION MSA	P-VALUE ORTHOLOGS CONSERVATION MSA	PARALOGS CONSERVATION MSA	P-VALUE PARALOGS CONSERVATION MSA	L-R COEVOLUTION MSA	P-VALUE L-R COEVOLUTION MSA	L-L COEVOLUTION MSA	P-VALUE L-L COEVOLUTION MSA	ORTHOLOGS CONSERVATION MSTA	P-VALUE ORTHOLOGS CONSERVATION MSTA	PARALOGS CONSERVATION MSTA	P-VALUE PARALOGS CONSERVATION MSTA	L-R COEVOLUTION MSTA	P-VALUE L-R COEVOLUTION MSTA	L-L COEVOLUTION MSTA	P-VALUE L-L COEVOLUTION MSTA	AVG DIR SCORE	P-VALUE SUM	P-VALUE RANK	MSA P-VALUE SUM
1	1N	0.55	0.58	0.25	0.36	0.18	0.34	0.56	0.81	0.59	0.33	0.27	0.42	0.24	0.02	0.24	0.05	0.53	2.90	5	2.09
2	2S	0.59	0.33	0.27	0.42	0.20	0.17	0.59	0.87	0.41	0.99	0.27	0.42	0.39	0.00	0.36	0.24	0.51	3.44	12	1.79
3	3D	0.53	0.70	0.15	0.11	0.20	0.17	0.59	0.87	0.26	1.00	0.27	0.42	0.57	0.00	0.47	0.57	0.51	3.84	24	1.85
4	4S	0.55	0.58	0.32	0.59	0.25	0.01	0.52	0.72	0.31	1.00	0.27	0.42	0.51	0.00	0.47	0.57	0.50	3.88	25	1.90
5	5E	0.51	0.80	0.25	0.36	0.21	0.11	0.52	0.72	0.53	0.70	0.28	0.45	0.18	0.34	0.58	0.85	0.48	4.33	45	1.98
6	6C	0.86	0.00	0.78	1.00	0.00	1.00	0.00	0.00	0.86	0.00	0.77	1.00	0.00	1.00	0.00	0.00	0.52	4.00	29	2.00
7	7P	0.57	0.45	0.31	0.55	0.16	0.55	0.53	0.74	0.61	0.22	0.40	0.82	0.18	0.34	0.46	0.54	0.48	4.21	43	2.30
8	8L	0.48	0.91	0.13	0.08	0.25	0.01	0.53	0.74	0.40	1.00	0.18	0.17	0.29	0.00	0.54	0.77	0.51	3.68	18	1.75
9	9S	0.56	0.52	0.30	0.52	0.18	0.34	0.47	0.57	0.56	0.52	0.27	0.42	0.18	0.34	0.53	0.74	0.49	3.96	27	1.94
10	10H	0.68	0.03	0.43	0.88	0.12	0.89	0.40	0.35	0.64	0.10	0.25	0.36	0.15	0.66	0.46	0.54	0.51	3.79	23	2.14
11	11D	0.53	0.70	0.26	0.39	0.25	0.01	0.47	0.57	0.57	0.45	0.23	0.29	0.18	0.34	0.58	0.85	0.50	3.60	15	1.67
12	12G	0.60	0.27	0.28	0.45	0.19	0.24	0.56	0.81	0.57	0.45	0.28	0.45	0.21	0.11	0.58	0.85	0.48	3.65	17	1.78
13	13Y	0.68	0.03	0.55	0.98	0.09	0.98	0.19	0.02	0.68	0.03	0.50	0.96	0.10	0.96	0.35	0.21	0.50	4.17	40	2.01
14	14C	0.86	0.00	0.78	1.00	0.00	1.00	0.00	0.00	0.86	0.00	0.78	1.00	0.00	1.00	0.00	0.00	0.52	4.00	30	2.00
15	15L	0.54	0.64	0.38	0.77	0.09	0.98	0.35	0.21	0.54	0.64	0.38	0.77	0.09	0.98	0.33	0.17	0.48	5.16	51	2.60
16	16H	0.70	0.01	0.57	0.99	0.10	0.96	0.35	0.21	0.70	0.01	0.57	0.99	0.10	0.96	0.24	0.05	0.48	4.19	41	2.18
17	17D	0.35	1.00	0.27	0.42	0.36	0.00	0.35	0.21	0.35	1.00	0.27	0.42	0.36	0.00	0.35	0.21	0.52	3.27	10	1.63
18	18G	0.66	0.06	0.61	1.00	0.00	1.00	0.00	0.00	0.66	0.06	0.61	1.00	0.00	1.00	0.00	0.00	0.51	4.11	37	2.05
19	19V	0.52	0.76	0.18	0.17	0.20	0.17	0.53	0.74	0.52	0.76	0.18	0.17	0.20	0.17	0.54	0.77	0.50	3.69	19	1.83
20	20C	0.86	0.00	0.78	1.00	0.00	1.00	0.00	0.00	0.86	0.00	0.78	1.00	0.00	1.00	0.00	0.00	0.52	4.00	30	2.00
21	21M	0.54	0.64	0.23	0.29	0.17	0.44	0.58	0.85	0.54	0.64	0.23	0.29	0.17	0.44	0.51	0.69	0.48	4.30	44	2.23
22	22Y	0.68	0.03	0.55	0.98	0.09	0.98	0.28	0.09	0.68	0.03	0.55	0.98	0.09	0.98	0.22	0.03	0.49	4.11	36	2.08
23	23I	0.53	0.70	0.37	0.74	0.19	0.24	0.46	0.54	0.53	0.70	0.37	0.74	0.19	0.24	0.36	0.24	0.49	4.15	38	2.22
24	24E	0.53	0.70	0.27	0.42	0.23	0.04	0.57	0.84	0.55	0.58	0.33	0.62	0.22	0.07	0.52	0.72	0.48	3.98	28	1.99
25	25A	0.52	0.76	0.24	0.32	0.27	0.00	0.46	0.54	0.53	0.70	0.19	0.19	0.25	0.01	0.45	0.50	0.53	3.02	9	1.62
26	26L	0.52	0.76	0.34	0.65	0.15	0.66	0.42	0.41	0.52	0.76	0.39	0.79	0.14	0.75	0.37	0.26	0.48	5.03	50	2.47
27	27D	0.51	0.80	0.34	0.65	0.30	0.00	0.42	0.41	0.51	0.80	0.34	0.65	0.30	0.00	0.43	0.44	0.51	3.76	22	1.86
28	28K	0.46	0.95	0.34	0.65	0.29	0.00	0.43	0.44	0.46	0.95	0.34	0.65	0.29	0.00	0.45	0.50	0.49	4.15	39	2.04
29	29Y	0.62	0.18	0.13	0.08	0.15	0.66	0.53	0.74	0.62	0.18	0.13	0.08	0.15	0.66	0.54	0.77	0.53	3.34	11	1.66
30	30A	0.54	0.64	0.30	0.52	0.12	0.89	0.53	0.74	0.54	0.64	0.30	0.52	0.12	0.89	0.53	0.74	0.46	5.59	53	2.80
31	31C	0.86	0.00	0.78	1.00	0.00	1.00	0.00	0.00	0.86	0.00	0.78	1.00	0.00	1.00	0.00	0.00	0.52	4.00	30	2.00
32	32N	0.51	0.80	0.18	0.17	0.22	0.07	0.43	0.44	0.51	0.80	0.18	0.17	0.22	0.07	0.45	0.50	0.53	3.02	8	1.48
33	33C	0.85	0.00	0.78	1.00	0.01	1.00	0.03	0.00	0.85	0.00	0.78	1.00	0.01	1.00	0.07	0.00	0.51	4.00	34	2.00
34	34V	0.55	0.58	0.13	0.08	0.24	0.02	0.53	0.74	0.55	0.58	0.13	0.08	0.24	0.02	0.55	0.79	0.53	2.90	4	1.43
35	35V	0.50	0.84	0.23	0.29	0.28	0.00	0.56	0.81	0.50	0.84	0.22	0.27	0.30	0.00	0.57	0.84	0.50	3.90	26	1.96
36	36G	0.61	0.22	0.53	0.98	0.09	0.98	0.20	0.02	0.62	0.18	0.45	0.91	0.10	0.96	0.38	0.29	0.48	4.53	48	2.20
37	37Y	0.70	0.01	0.67	1.00	0.10	0.96	0.08	0.00	0.65	0.08	0.59	0.99	0.16	0.55	0.22	0.03	0.51	3.63	16	1.97
38	38I	0.52	0.76	0.29	0.49	0.27	0.00	0.50	0.66	0.56	0.52	0.24	0.32	0.22	0.07	0.55	0.79	0.50	3.60	14	1.90
39	39G	0.66	0.06	0.61	1.00	0.01	1.00	0.02	0.00	0.63	0.14	0.55	0.98	0.04	1.00	0.20	0.02	0.50	4.20	42	2.05
40	40E	0.53	0.70	0.37	0.74	0.18	0.34	0.48	0.60	0.54	0.64	0.40	0.82	0.18	0.34	0.34	0.19	0.48	4.36	46	2.38
41	41R	0.60	0.27	0.55	0.98	0.01	1.00	0.02	0.00	0.60	0.27	0.54	0.98	0.01	1.00	0.06	0.00	0.51	4.51	47	2.26
42	42C	0.86	0.00	0.78	1.00	0.01	1.00	0.02	0.00	0.86	0.00	0.77	1.00	0.01	1.00	0.06	0.00	0.51	4.00	33	2.00
43	43Q	0.61	0.22	0.32	0.59	0.12	0.89	0.45	0.50	0.61	0.22	0.31	0.55	0.12	0.89	0.57	0.84	0.48	4.70	49	2.20
44	44Y	0.60	0.27	0.26	0.39	0.20	0.17	0.56	0.81	0.66	0.06	0.26	0.39	0.16	0.55	0.57	0.84	0.50	3.47	13	1.64
45	45R	0.51	0.80	0.18	0.17	0.22	0.07	0.57	0.84	0.51	0.80	0.26	0.39	0.24	0.02	0.50	0.66	0.50	3.74	21	1.87
46	46D	0.56	0.52	0.34	0.65	0.20	0.17	0.53	0.74	0.54	0.64	0.29	0.49	0.22	0.07	0.55	0.79	0.48	4.07	35	2.08
47	47L	0.52	0.76	0.48	0.94	0.08	0.99	0.06	0.00	0.52	0.76	0.47	0.93	0.06	1.00	0.06	0.00	0.51	5.37	52	2.69
48	48K	0.55	0.58	0.24	0.32	0.22	0.07	0.50	0.66	0.53	0.70	0.22	0.27	0.23	0.04	0.38	0.29	0.52	2.92	6	1.63
49	49W	0.66	0.06	0.15	0.11	0.13	0.83	0.56	0.81	0.93	0.00	0.20	0.21	0.13	0.83	0.10	0.00	0.60	2.86	3	1.81
50	50W	0.73	0.00	0.17	0.15	0.20	0.17	0.53	0.74	0.89	0.00	0.17	0.15	0.15	0.66	0.15	0.01	0.62	1.87	1	1.06
51	51E	0.53	0.70	0.26	0.39	0.17	0.44	0.56	0.81	0.56	0.52	0.27	0.42	0.17	0.44	0.14	0.01	0.52	3.73	20	2.34

**52**	52L	0.54	0.64	0.20	0.21	0.23	0.04	0.53	0.74	0.48	0.91	0.27	0.42	0.25	0.01	0.19	0.02	0.54	3.00	7	1.64
**53**	53R	0.55	0.58	0.15	0.11	0.21	0.11	0.57	0.84	0.53	0.70	0.27	0.42	0.25	0.01	0.26	0.06	0.54	2.83	2	1.64

Table S2. EGF mutants relative ranking for the DIRpred score. The four partial scores were ranked from 1st to 55th on each protein site, for the two methods using either the Multiple Sequence Alignment (MSA) or the Multiple Structure Alignment (MSTA). The ranking of the four sites chosen for mutagenesis are reported.

Asn-32:

Score	*I*	*II*	*III*	*IV*
MSA	46	8	13	21
MSTA	44	5	17	31

Asp-46:

Score	*I*	*II*	*III*	*IV*
MSA	24	31	18	33
MSTA	32	28	17	46

Lys-48:

Score	*I*	*II*	*III*	*IV*
MSA	26	14	13	29
MSTA	37	9	15	27

Trp-50:

Score	*I*	*II*	*III*	*IV*
MSA	7	7	18	33
MSTA	2	3	33	12

Table S3. ANOVA details. The full results of the ANOVA analyses. When not specified, the row factor represent the times of dosage for the treatment, while the column factor represent the ligands used in the treatment.

**Figure 4A**					
**Source of Variation**	**% of total variation**	**P value**	**P value summary**	**Significant?**	
Interaction	1.786	0.6222	ns	No	
Row Factor	0.3636	0.4439	ns	No	
Column Factor	83.23	<0.0001	****	Yes	
**ANOVA table**	**SS (Type III)**	**DF**	**MS**	**F (DFn, DFd)**	**P value**
Interaction	0.3996	10	0.03996	F (10, 66) = 0.8075	P=0.6222
Row Factor	0.08137	2	0.04068	F (2, 66) = 0.8222	P=0.4439
Column Factor	18.62	5	3.725	F (5, 66) = 75.28	P<0.0001
Residual	3.266	66	0.04948		
**Figure 4B**					
**ANOVA table**	**SS**	**DF**	**MS**	**F (DFn, DFd)**	**P value**
Treatment (between columns)	0.3553	5	0.07105	F (5, 18) = 18.52	P<0.0001
Residual (within columns)	0.06907	18	0.003837		
Total	0.4243	23			

Figure 5A_Bj					
**Source of Variation**	**% of total variation**	**P value**	**P value summary**	**Significant?**	
Interaction	15.49	0.0153	*	Yes	
Row Factor	1.694	0.2017	ns	No	
Column Factor	74.11	<0.0001	****	Yes	
**ANOVA table**	**SS**	**DF**	**MS**	**F (DFn, DFd)**	**P value**
Interaction	3523	10	352.3	F (10, 18) = 3.204	P=0.0153
Row Factor	385.4	2	192.7	F (2, 18) = 1.752	P=0.2017
Column Factor	16857	5	3371	F (5, 18) = 30.66	P<0.0001
Residual	1980	18	110		

Figure 5B_A431					
**ANOVA table**	**SS**	**DF**	**MS**	**F (DFn, DFd)**	**P value**
Treatment (between columns)	10783	5	2157	F (5, 6) = 4.811	P=0.0410
Residual (within columns)	2690	6	448.3		
Total	13472	11			

Figure S7_EGFR					
**Source of Variation**	**% of total variation**	**P value**	**P value summary**	**Significant?**	
Interaction	20.35	<0.0001	****	Yes	
Row Factor	3.707	0.0039	**	Yes	
Column Factor	51.29	<0.0001	****	Yes	
**ANOVA table**	**SS**	**DF**	**MS**	**F (DFn, DFd)**	**P value**
Interaction	5.736	5	1.147	F (5, 60) = 9.908	P<0.0001
Row Factor	1.045	1	1.045	F (1, 60) = 9.022	P=0.0039
Column Factor	14.45	5	2.89	F (5, 60) = 24.96	P<0.0001
Residual	6.947	60	0.1158		

Figure S7_pEGFR					
**Source of Variation**	**% of total variation**	**P value**	**P value summary**	**Significant?**	
Interaction	0.968	0.8468	ns	No	
Row Factor	0.04965	0.75	ns	No	
Column Factor	71.87	<0.0001	****	Yes	
**ANOVA table**	**SS (Type III)**	**DF**	**MS**	**F (DFn, DFd)**	**P value**
Interaction	0.1029	5	0.02057	F (5, 56) = 0.3999	P=0.8468
Row Factor	0.005277	1	0.005277	F (1, 56) = 0.1026	P=0.7500
Column Factor	7.637	5	1.527	F (5, 56) = 29.69	P<0.0001
Residual	2.881	56	0.05144		

Figure S8_Bj					
**Source of Variation**	**% of total variation**	**P value**	**P value summary**	**Significant?**	
Interaction	17.94	0.0005	***	Yes	
Row Factor	11.91	<0.0001	****	Yes	
Column Factor	64.75	<0.0001	****	Yes	
**ANOVA table**	**SS**	**DF**	**MS**	**F (DFn, DFd)**	**P value**
Interaction	6438	10	643.8	F (10, 18) = 5.982	P=0.0005
Row Factor	4273	2	2136	F (2, 18) = 19.85	P<0.0001
Column Factor	23236	5	4647	F (5, 18) = 43.18	P<0.0001
Residual	1937	18	107.6		

Figure S8_SA24					
**Source of Variation**	**% of total variation**	**P value**	**P value summary**	**Significant?**	
Interaction	10.6	0.6407	ns	No	
Row Factor	4.055	0.248	ns	No	
Column Factor	61.15	0.0002	***	Yes	
**ANOVA table**	**SS**	**DF**	**MS**	**F (DFn, DFd)**	**P value**
Interaction	2103	10	210.3	F (10, 18) = 0.7881	P=0.6407
Row Factor	804.8	2	402.4	F (2, 18) = 1.508	P=0.2480
Column Factor	12137	5	2427	F (5, 18) = 9.097	P=0.0002
Residual	4803	18	266.8		

Figure S8_SA48					
**Source of Variation**	**% of total variation**	**P value**	**P value summary**	**Significant?**	
Interaction	5.055	0.8368	ns	No	
Row Factor	2.164	0.3345	ns	No	
Column Factor	76.06	<0.0001	****	Yes	
**ANOVA table**	**SS**	**DF**	**MS**	**F (DFn, DFd)**	**P value**
Interaction	1464	10	146.4	F (10, 18) = 0.5440	P=0.8368
Row Factor	626.6	2	313.3	F (2, 18) = 1.164	P=0.3345
Column Factor	22023	5	4405	F (5, 18) = 16.37	P<0.0001
Residual	4843	18	269.1		

Supplementary data 1. Time-lapse video of the proliferation of A431 and Bj-5tα cells. Treatment with 100 nM ligand was performed at the 24h mark. Evidence of a balance between differentiation and proliferation are found in the fibroblast, where a reduced proliferation is associated to cellular morphology differences in WT and K48T.
